# The Mental Homologies of Mammals. Towards an Understanding of Another Mammals World View

**DOI:** 10.3390/ani7120087

**Published:** 2017-11-23

**Authors:** Marthe Kiley-Worthington

**Affiliations:** Centre d’Eco-Etho Recherche et Education, La Combe Benzaudun sur Bine, 26460 Drôme, France; marthekileyworthington@gmail.com; Tel.: +33-47-553-2027

**Keywords:** animal cognition, theory of mind, animal epistemology, conditional anthropomorphism

## Abstract

**Simple Summary:**

Mammals are defined by their similarities in bodies, behaviours and minds, where the mind is defined as the organized totality of mental processes. Mental similarities between related species, known as homologies, have largely been ignored by cognitive scientists since Darwin. Today, some behavioural scientists hold a series of beliefs which do not recognize them. However, people who have to do with animals daily have always recognized common mental traits they have with their animals. After 25 years of multi-disciplinary study, nine important mammalian mental homologies have been delineated: (1) All mammals have innate behaviour tendencies which are molded by the individual’s lifetime experiences. (2) They are sentient (that is they feel and have emotions) and consequently are conscious: awake and aware of the world. (3) They all learn in similar ways. (4) They all have to acquire ecological knowledge to live and reproduce. (5) They acquire social knowledge, have a social contract and can develop different traditions and cultures. (6) They have to know about others’ intentions to be social, that is they all have a “theory of mind”: an awareness that others have body/mind beings. (7) They are aware of their own body and feelings, thus they must be self-aware. (8) They are moral agents because they know when they have obeyed the social contract and when not. (9) They have a simple aesthetic sense because they like some things and dislike others. Sentience appears to be the power house behind these and other mental aptitudes which follow from them such as beliefs, decisions, episodic memory, imagination and mental time travel. In order to compare the “beings” (body-mine wholes) of different species and their world views, Conditional Anthropomorphism is proposed. This recognizes our common mental traits (anthropomorphism) takes into account different species specialties and examines the past experience of that individual to indicate how his/her world view is molded by lifetime experiences (conditional). Such studies are essential to improve their welfare and enriching our lives.

**Abstract:**

Mammals’ mental homologies include that they look after their young, suckle and protect them; they acquire information about the world by learning. They have five types of sensory receptors and a brain to analyze the information and they feel: that is they are sentient. Mental homologies have been largely ignored by behavioural scientists since Darwin because of certain historical beliefs. This however has not been the case for people who have had to do with non-human mammals who have long recognized their mental similarities to humans. As a result, behavioural science has sponsored some inappropriate research (examples are given). The study of another mammal species epistemology, (knowledge and world view) requires a recognition of these mental homologies. The result of a 25 year multi-disciplinary study indicates that there are nine mammalian mental homologies which define mammals. These are discussed and reviewed and further mental aptitudes which logically follow from these are pointed out. A Conditional Anthropomorphic approach is proposed. By recognizing the body/mind, whole “being” homologies of mammals, we can advance in understanding other mammal species’ and individual’s epistemology (world view), and consequently better their welfare and enrich our own lives.

## 1. Introduction

Species are classified as a result of cladistics (Table 1 and Schuh et al., 2009 [1]) and Linnean systematics (Table 1., Grove and Newell 1942 and Young 1950 [2,3]). Today the term “synapomorphy” (Table 1) is also used to indicate a characteristic shared by evolutionary descendants. Whatever term is used, it is the similarities that define a body/mind being as a “vertebrate”, a “reptile” or a “mammal” (Hegel 1807, Grove and Newell 1942, Young 1950 and Hennig [2,3,4,5]). It is possible that these body-mind homologies are present in other vertebrates, however, this discussion is confined to mammals.

Placental mammals are defined as having an endo-skeleton with recognizably similar bones, internal organs and physiology. They are warm blooded (homeothermic Table 1) and consequently must make decisions to keep themselves warm or cool. To live and reproduce in different habitats and seasons, each individual has to learn about their environment (Kiley-Worthington [8]) and all mammals acquire information by silent learning and learn voluntarily which involves acquiring information by making choices and decisions (Pearce 2008 [16] and Dickinson [17]) although the knowledge they acquire may differ. For example, all male mammals have to learn to court and copulate; all females to suckle and look after their infants. As a result, all mammals have the basis of a social life (Midgley [18]). To have a social life with organization and rules, they have to be aware that others have intentions and desires, consequently, all mammals must have a “theory of mind” (Table 1).

These definitions have been adapted from the Oxford English dictionary (Drever [19] and Flew, 1979 [20]), except where otherwise mentioned. Most of these terms are in general use therefore it is important to define how they are used. Where they have been invented by cognitive ethologists, the original or a current reference is given. They are used in line with these desfinitions to allow a careful investigation of mammalian mental homologies. 

Mammals have five types of sensory receptors with which to perceive the world, which function similarly but are also specialized for the particular needs of that species. They all have a brain to which information from the sense organs is sent, analyzed and acted on and all mammals are sentient, that is they feel & have emotions (Damasio 1994 [21], Mendl et al. [22] and Anderson & Adolphs [23]).

If we want to advance in understanding “what it is to be another mammal”, that is their united body mind “being”, world view, knowledge of the world: their epistemology (von Uexhall [24,25]) first, we must recognize the homologies of their bodies and their minds which define them as a mammal. Superimposed on these general mammalian characteristics will be the particular species characteristics of body and mind. The third step is to recognize the individual’s particular differences to other members of his species as a result of lifetime experiences. Here, we address the common mental characteristics of mammals: what mental attributes define mammals. 

A recognition of sentience acknowledges that non-human mammals must have a “mind” since to feel, there must be something to feel with. Both Darwin [26], and James [27] recognized similarities in emotions or sentience of related species, but this was largely ignored by scientists for a century. 

By contrast, the public recognition of human and non-human mammals’ mental similarities has its origins in centuries of contact with other mammals who were raised, handled, taught, helped in wars, worked on farms, used for transport, for entertainment and as companions and friends living by human firesides (Rollin [28]). Such knowledge (in the days when the majority of humans lived in rural environments) was called “common sense”, today it has been called “folk knowledge” (as distinct from folk belief, Table 1). To teach a bullock to pull a cart, a cow to be milked, a horse to be ridden or go to war, a dog to work sheep or elephants to move timber around, and a host of others skills, it is essential to recognize that other mammals have many mind-skills similar to their human handlers. Without recognizing this, the handlers could not teach or use them as they do, even though this has not always been to the animals’ benefit any more than humans using other humans has been. 

Therefore, unlike scientists, the general public did not ignore the presence of non-human mammalian minds and emotions, and consequently, their ability to suffer, even during the 19th century. The first animal protection law which recognized that cattle could suffer was passed in 1822 in the UK. Anna Sewell’s book Black Beauty (1877) [29] which described the suffering of horses used for transport, expanded the public’s interest and helped to stimulate further changes in the law recognizing animal suffering in 1911 in the UK.

Thus, long before “ethology” or “comparative psychology” was invented, humans recognized similar mental aptitudes, that is mental homologies, between themselves and non-humans. Today, this folk-knowledge (Table 1) is often lacking in comparative cognitive scientists as well as the general public as a result of their urban backgrounds where the majority have no or very little daily experience with animals. What was “common sense knowledge” is now often the subject of expensive experimental tests, even though everyone who has had to do with that species knows he has the mind skills being tested because without them, they could not do what they do with the animals and have done for centuries. There is also a host of literature pointing out non-human mammals’ mental attributes from Xenophon (350 BC) to the present. But, it is equally important to be aware of preconceptions (folk beliefs, Table 1) which both those who live with their animals and scientists may hold. 

A brief summary of the history of ideas and beliefs (Table 1) concerning animal behaviour and mentality will help us to understand why mammalian mental homologies are still not recognized by many scientists. There are many recent examples in both animal welfare science and cognitive ethology journals which illustrate this. For example, can horses recognize each others’ calls? (Wathan et al. [30]), or human facial expressions (Smith et al. [31]). Today, behavioural researchers who have little or no knowledge of the species they are researching, receive funding and acclaim when they “prove” such known facts (de Waal [32] for further examples in primates). But, those who deal with horses or dogs every day could not teach them the way they do unless they used these mental abilities (Podjansky 1967 [33] and Mabbutt [34], and many others). Why, since horses recognize each other and are skilled visual communicators (established in 1907 by Clever Hans: Candland [35]) would they not be able to recognize others’ calls, or respond to intentions of humans by observing their facial expressions? Such “proofs” do not add to our knowledge. 

Once sentience (Table 1) was finally recognized by scientists, another reason for justifying human superiority in mental attributes immerged. In the 1950–1970’s speaking and understanding human language was believed to be only possible for humans and quickly became the reason why it was believed only humans had a host of mental abilities (e.g., Chomsky [36], Pinter [37], Gazzinga [38] and Marvizon 2016 [39]). Since then various primates have learnt to comprehend, and even use human-type language by using sign language and computers (review Fouts [40] and Savage-Rumbaugh [41]) dogs, horses and elephants have been known to learn the meaning of words, phrases and sentences for some centuries (Johnson [42] and Kiley-Worthington & Randle [43]).

The study of learning by experimental psychologists did not undermine the belief that non-human behaviour was controlled genetically, that is in a sense robotically, because for the first half of the 20th century, learning was described as a type of neo-instinctive “conditioning” which allowed sentience, feeling or having emotions, as well other related mental attributes to be swept aside and dubbed “motivation”, (e.g., Watson [44], Skinner [45], and the subsequent field of experimental psychologists). The common person would call “motivation” (Table 1): “wanting”, undeniably a “feeling”. 

The result of the behaviourists’ efforts was that all animals in science were often (and still are sometimes) delegated to robotic status who behave because of their genes and instincts. This gave rise to the study of “ethology” (e.g., Tinbergen [46] and Lorenz [47]) and later to socio-biology (e.g., Wilson 1975 [12]). Everyday, proximal behaviour, it’s mammalian commonalities, flexibility, and emotionality were ignored, or labeled “anthropomorphism”, (an unscientific explanation, despite Darwin’s claim), except by a courageous few (Midgley [18], von Uexhull [25], Savage-Rumbaugh [41], Griffin [48], Fisher [49] and Burghardt 1990 [50]). In 1969 when examined for a PhD on the cause and function of mammalian communication: it was not acceptable to say that “the dog was frightened”. It had to be rephrased: “the dog behaves “as if” he was frightened”. This robotic quality of non-human mammals persists with computer models of non-human behaviour building (e.g., Kappeler, et al., 2013 [51]), which are notably lacking for humans.

In the second half of the 20th century as the use of animals in agriculture and research grew, more people became urban and had less to do with animals and skepticism about animals’ minds grew. As a result, both scientists and the public adopted a series of “beliefs of convenience” whereby it was socially acceptable to distance themselves from the profound moral issues emerging in their work or living. An example of this is the development of some farm animal factories where the animals suffered all their lives (Harrison [52] and Carson [53]). Scientists were equally to blame, for example, there continued to cause lab animals pain and suffering and there was no postsurgical use of analgesic in the US until 1985 (US Department of Agriculture [54]), even though the law had recognized they feel and suffer. This lack of recognition of the common mental homologies that define mammals led to “beliefs of convenience” where suffering of animals was not a moral issue as long as it advances humans social status or money earning. Today, the moral issues are difficult to avoid, but because common mental homologies have still not been recognized by scientists, there is some unproductive or hair-splitting research that has continued for decades (e.g., Premack & Woodruff [15], Terrace and Metcalfe 2005 [55]).

As a result, scientists involved in behavioural sciences and animal welfare often have a series of beliefs that hamper an admittance of our common mammalian mental homologies and discourage advancement in mammalian epistemology. These are:

(1) It is believed that “good research” on mental attributes of a species, must be conducted from a tabula-rasa position that is, until it is proved that this species can do this mental task, it cannot be assumed, even though it is a mammal who by definition has this skill (e.g., to feel or to learn). This raises the question of what needs “proof”. An example of a discussion 30 years ago in Glasgow (Applied Philosophy Society, Glasgow Vet school 1987), was whether it was necessary to give ducks water (not to drink, but to swim in in their enclosure). Logic dictates that a duck is, by its nature, that is by definition, a water bird, therefore it requires water, not just to drink but to swim on, bathe in, feed in etc. This is not something that needs to be tested, unless it is necessary to test whether the subject is, in fact, a duck! Similarly, a gnu is a plains living social quadruped who is a sophisticated visual communicator, runs fast and leaps and jumps around. Because he is who he is, a normal gnu requires to be able to do these things. It is not necessary to test this, any more than it is necessary to prove that a normal baby must have the opportunity to learn to walk on two legs.
(2) Conclusions are rapidly drawn on the mental inabilities of non-human mammals as the result of:
  (a) *Negative experimental results*. If that animal does not perform in that set of experimental conditions, it is concluded that the individual or the species, is incapable of that performance, but, this may be the result of a whole range of experimental conditions (see de Waal [32] for critical review).
  (b) *Inappropriate tests*. Different species may see, hear, feel, smell, taste or manipulated things others do not. This must be properly understood before doing tests. For example, “self awareness” tests with a mirror concluded that as a result of an elephant’s performance, the elephant was not self aware (Povinelli [56]). On examination of these experiments, it is clear (i) that the elephant could not see himself in the mirror (de Waal [32]); (ii) the elephant used the mirror image to manipulate objects with his trunk, demonstrating he was aware that his trunk was his and that it had to be manipulated the reverse way in the mirror image, something humans find difficult (Kiley-Worthington [8]).
(3) Assumptions concerning mental attributes are often made from a duelist position. This is the separation of the body from the mind as opposed to monism: the belief in the interaction of body and mind combining in the living “being” (Table 1). The researchers admit that non-human mammals have similar bodies, but they cannot accept that they have similarities of minds (e.g., Shettleworth [57] and others), thus separating them. Duelism is *not* in line with evolutionary theory, but rather with special creationism and human theology and has long been debated (e.g., Hegel [4], Damasio [21], Spinoza [58], Heidegger [59], Wittgenstein [60] among others). It has been rightly argued that animal welfare scientists lack a grounding in ethics (Rollin [28] and Zemanova [61]) which, to contribute to this science, they need. Comparative psychologists appear to lack a grounding in philosophy of mind and, perhaps logic! For example, although believing that non-human mammals are sentient, the comparative psychologists deny them consciousness, but, they feel with something; feelings are felt by an awake conscious being, and this needs a mind (Table 1).
(4) Some researchers must also believe that non-human mammals are also duelists. “*Do non humans have a theory of mind*”? (Premack and Woodruff [15], Terrasse & Metcalf [55] many others). This question assumes that the non-human mammal recognizes that another has a body, but not that “the other” is alive, behaving and feeling that s/he has intentions, makes decisions and choices that is must have a mind. If this is the case, all non-human mammals are either robots (with no mental abilities so they do not feel, learn or recognize another is alive) or they are duelists. It seems unlikely that rats, elephants, dolphins, not to mention dogs, cattle and horses are all either robots or duelists.
  If a mammal has any social life it implies some social organization [62]. To have this, s/he must recognize others and be aware of others’ intentions, in other words that others have feelings shown by their intentions, that is recognizing another as a combined body-mind being.
(5) It is often assumed that humans are mentally superior to all other species. (Gazzinga [38] and Malcomb [63]). But, it is evident that, for example, humans’ sensory receptors and the resulting mental assessments are not always “superior” to those of other species; there are many non-human mammals who smell, feel, taste, see, or hear better and differently from humans and consequently, have some possibly “superior” and certainly “different” knowledge, feelings and assessments of the world around them (see Plotnik [64]). Humans have superior verbal skills, but this does not mean that only they can think (Pinter [37]), or that only they have ideas of the past and future. The interesting question is not “do they think?” but “what do they think about?” (Lea & Kiley-Worthington [65] and Morell [66]). If the belief in humans’ superiority in all mental skills is maintained, then why and how has this evolutionary leap occurred? Recent assessments of Synthetic Theory including molecular theory, indicate that evolutionary leaps are unlikely (Futuyma 2015 [67]).
  Popular science underlines the belief in the overall mental superiority of humans and the uniqueness of their cooperation. But, cooperation in non-humans is well known (Dugatkin [68]). Do humans actually show superior empathy, altruism, and cooperation? The history of human civilization, past and present, shows rather the reverse. Today, where many starve, while others eat too much and parts of this “cooperative/empathetic/civilized society” continues to kill and rape men, women and children by the thousand [69]. This does not occur in other mammalian societies. Even when things are difficult and resources scarce, non-human mammals, generally obey the social contract, one of whose rules is not to kill members of your own species. It would seem more likely that human “civilization” has it’s origin in primate competitive hierarchies which have lead to an increasing interest in a desire for power, rather than to greater cooperation with each other, or the environment.
(6) The mental consequences of sentience are not recognized. If non-humans have feelings and feel them, then they are *conscious of that feeling*, and consequently are *conscious of being in the world.* Sentience and consciousness of being in the world indicates that they have a subjectivity (Table 1): they are subjects with a point of view and an epistemology (Table 1 knowledge and experiences).

Because of scientific skepticism, there was no consensus of belief that non-human mammals had minds by scientists until the Cambridge conference in 2012. This, belief, coupled with unwarranted assumptions made by some investigators even in the light of recent evidence concerning the recognition of types of mammalian minds and their homologies, allows both scientists and the general public to distance themselves from moral issues and enables beliefs of convenience (see above). To progress in an understanding of others’ mentality, and how to better their welfare, these beliefs need serious examination. The first step is to recognize what defines a mammalian being: an interactive mammalian body/mind. 

## 2. Method

This investigation reviews, amalgamates and critically assesses information from any relevant source: evolutionary biology and systematics, physiology, anatomy, neurophysiology, environmental science and ethics, observational and experimental behavioural and cognitive sciences including detailed observational and experimental research, philosophy of mind, folk knowledge and experiences, an examination of ones’ own feelings/emotional responses in similar situations with those of non-human mammals and, finally, detailed one to one research into the teaching of different mammalian species (Kiley-Worthington & Rendle-Worthington [70]). 

## 3. Results and Discussion of the Mental Homologies of Mammals

The result of this research spanning some 25 years, are the following mammalian mental homologies discussed in turn. 

### 3.1. Innate Tendencies

Innate/Instinctive tendencies recognize how instinct and learning can unite to increase the knowledge and adaptability of the mammal. Von Uexkull [25] and later Thorpe [71] tried to amalgamate learning and instinct, but were largely ignored because ethology’s secure belief was in the central role of instincts controlling non-human behaviour (Tinbergen [46] and Lorenz [47]). This subsequently gave rise to socio-biology (Wilson 1975 [12]) and became a “meme” (Dawkins [72]) for research ethologists and evolutionary biologists from 1969 to around 2000. Evolutionary behaviourist’s dogma of the later part of the 20th century, was backed by theorists, computer models, mathematics (e.g., Maynard-Smith [73]), and the idea of the “selfish-gene” (Dawkins [72]). The explanations of behaviour concentrated on evolutionary genetic constraints, ignoring the mind and knowledge acquired with the help of feelings that control most day to day behaviour. 

Studies of learning followed a similar pattern. Pavlov [74] described “Conditioning” as the establishment of a Conditioned Response that resembled an inflexible learnt instinct. As a result, the behaviourists swept emotions and feelings aside to explain learning by the establishment of simple inflexible rules (e.g., Skinner [45]). 

Over the next half century, some important discoveries were made concerning both instincts and learning in mammals, but their erratic, flexible, everyday behaviour was generally ignored. To confuse the issues further, elaborate jargons were developed which successfully cloak experimental psychologists’ and ethologists’ work in mystery, although “science” is supposed to deal in knowledge and facts, not mystery (further discussion Midgley [75])!

At the beginning of the 21st century, both instinct (inflexible innately programmed behaviours) and learning (flexible emotionally driven behaviours) were recognised as involved in almost all human and non-human mammalian behaviours. The mammal has “instinctive tendencies” to do particular things as a result of the innate species predispositions, but *when*, *whether*, *if, where*, *how s/he does this*, *is moulded by his/her lifetime experiences* of his body/mind being. For example, humans have an” instinctive tendency” to walk on two legs, but if, when, where, whether and how they learn to do this, is the result of their lifetime experiences. If they have a damaged leg, they may hop, if raise by wolves, crawl (Candland [35]). Bison, buffalo and cattle, have an “instinctive tendency” to live in multi-male troops (Reinhart & Reinhart [76] and Prins [77]), but whether they do or not, depends on their lifetime experiences including habitat, food resources and so on. Bulls raised alone, when introduced to others, may choose not to live in multi-male groups, and may separate themselves from cows [77] Although all mammals have innate tendencies to mate, different species have different courting rituals, these they learn by being aware of the females’ intentions and how and when to approach her. If they have not had the opportunity to learn this, they make mistakes and will be unsuccessful or injured. For example injuries of both stallion and mares are higher where neither have learnt socially how to court or mate. This has lead to the raping of mares (physically and chemically) in modern horse breeding, rather than allowing stallions and mares, to learn the sexual contract (Kiley-Worthington [9]).

One of the greatest evolutionary strengths of mammals is that much of their behaviour is genetically incorporated only as soft wired “tendencies” which can be moulded by lifetime experiences allowing the individual to acquire knowledge about the environment and adapt to it (Ewer [78], Thurfjell et al. [79], Buss [80] and Dickens, WT & Cohen, [81]). For example, humans and other domestic mammals such as cattle, sheep, horses and goats can adapt to live very different lives in different habitats, and wild mammals are learning to adapt and thrive in human urban environments (e.g., foxes and bears). When raised in cities, these will have very different knowledge from their rural cousins. When elephant orphans raised with humans, are put back in “the wild” or “nature reserves”, this can be a death sentence as they migrate back to human settlements and cause fear and damage to humans, resulting in being shot/murdered/culled [82]. This learning about different ecology and social norms, results in the same species having different cultures and traditions in different areas (Avital & Jablonka [82], Bonner 1980 [83]).

To summarise: mammalian behaviour is genetically programmed to the extent that every mammal has “instinctive tendencies” to do various things: (i) characteristic of mammals and (ii) of that species. But if, *how*, *where*, *when*, *whether and what they do*, *is moulded by their lifetime experiences*: *learning*. They have to acquire knowledge about all aspects of their environment ([8]). There has been some recognition of the importance of acquiring social knowledge by non-human mammals and birds (e.g., Shettleworth [57], de Waal & Bonnie [84]), but the amount and type of eco-ethological knowledge each individual of each species must acquire to live and reproduce, has received less attention (see Kiley-Worthington. 2000 & [85] for review).

### 3.2. Mammals Are Sentient and Therefore Conscious of Being in the World

Other mental attributes follow on logically from sentience. Firstly, if an alive being (which consists of a mind and a body) feels, then it is conscious of the feeling. “Conscious” is used here in the common sense meaning: awake and aware of what is around (Table 1) thus, a feeling mammal is “conscious”; that is awake and aware of the world around him, a basic mental aptitude of all normal mammals (Harnad 2012 [62,86]). If, when used by cognitive scientists or philosophers, it means something else, another word should be used to avoid confusion. There may be different levels or types, but all us normal awake mammals, are conscious of being in the world. When “unconscious”, we are not awake and aware of the world and may not feel. It is likely that all mammals, like humans, have behaviour that is affected by “unconscious” or “subconscious” memories or experiences. This is an interesting subject for future research. But, assuming that non-humans are not conscious that is “unconsciousness” of the everyday world in which they live is false, if they feel.

Secondly, mammals have a variety of feelings or emotions in common: pain, fear, hot, cold, anger, happiness/pleasure, excitement, among others (e.g., Prinz [87]). Every normal mammal can identify something like these emotional states in members of their own species. Many are also able to interpret the emotional state of another species of mammal, humans for example. Domestic mammals read ours and each others’ emotions or feelings which is why they can live with us. Tests conducted with urban humans who had never met pigs, found that even these people were successful in recognizing what the pigs were probably feeling (Welmeslsfelder 2008 [88]). This is because we have many common behaviours to express these emotions (e.g., running away when afraid, approaching cautiously when curious, exposing weapons (teeth, tools, feet, trunks, etc.) when angry, and a host of movements that indicate uncertainty or frustration (e.g., scratching, head shaking, yawning and many other behaviours performed more frequently in an approach/avoiding or frustrating situations (Ewer [78] and Bindra [89]). Excitement or arousal is also easily recognized (e.g., rushing around, leaping about, vocalising Kiley-Worthington 1969 [90]). These emotions all mammals feel similarly, because they are mammals, and so they can be identified between species. Without being able to interpret each others’ emotional states, the normal association of mammals would not be possible. 

But some species probably feel some emotions that other species cannot read so easily. This may be the result of their sensory systems, special bodies or special abilities. For example, a bat’s feeling when locating his prey by sonar; or the “snake face” of the stallion herding his own mares, or the feeling induced by smelling/touching/manipulating that an elephant must have with his trunk which combines senses and acts as a combined nose/mouth/hand. But, even here, we mammals have enough in common to be able to make an informed guess as to what that feeling might be like (Kiley-Worthington [9]).

What exactly each feeling feels like may never be known by another, either of the same or a different species because it is a private feeling: my red may not spark the same feelings as when you see the same red (e.g., Humphrey 2009 [91]). This “private world” is the “hard problem of the conscious mind” (Chalmers [92]). But, nevertheless our commonality ensures that we can read from the other that s/he is feeling “something like” I would feel in that situation, even if we do not experience exactly the same feeling. As a result we can live together with our own and other species and have either mono- or multi-species societies that have rules. 

Sometimes the signs of different feelings in different mammals are so subtle that we cannot pick up the cues as our receptors are inadequate, such as many smells or tastes for humans. Human verbalizing can also be a handicap because it often takes priority and causes lack of attention or awareness to other cues such as visual, smell or taste. But, humans can learn to observe, smell, taste, feel and even hear non-verbal descriptions, just like the non-verbal dog/horse/elephant/lion who with close contact, can learn to listen to and interpret much of human language (e.g., Reiss et al. [93]) as well as read the intentions of the human better than humans can. 

Whatever the subtleties of feelings, it is because mammals are able to read something about the feelings of others that they have, through evolution, been able to associate together or know when to avoid each other. It may also be why as a general rule we find it more difficult to get along with fish, reptiles and insects who may demonstrate their emotions differently, or have different ones. 

Communication conveys something about what the communicant is feeling to the recipient and what he is likely to do next. The recipient reads the communicator’s desires, as a result. All normal mammals convey “something about” their feelings to others which is understood by the recipient and acted on, consequently they all have a “theory of mind” (see below).

It is these characteristic feelings or emotions which may be the powerhouse behind many of the other mammalian mental abilities (Goleman [94] below).

### 3.3. All Mammals Learn

Perhaps the greatest mammalian evolutionary achievement is the sophistication of their learning which allows flexible behaviour and the acquisition of an enormous amount of knowledge. This knowledge or information is acquired by having the “motivation”, that is “wanting” to learn what ever is needed, so the driving force behind all learning is also a feeling or an emotion.

Learning comes in several types, Pavlovian Conditioning, Instrumental or Operant Conditioning, Associative, Social or Observational learning and Silent or Cognitive learning, (acquiring information without reinforcements) (e.g., Pearce [16] and Dickinson [17]). The ways things are learnt in the real world, unlike in laboratory experiments (where the central idea is to cut down variables), is generally a mixture of these.

The most important types of learning for the acquisition of knowledge are silent or latent learning, and voluntary instrumental or operant learning. Latent or silent learning is acquiring knowledge without any reinforcement, such as learning the geography of the home area, or knowing when changes have been made in the environment which make no particular difference to the individual’s life. For example, one of our mares walked past a log at the side of the road every day and took no notice of it. We changed its angle by 20 degrees and the next time she walked past she stopped, stared, pricked her ears and snorted at it: she had recognized that its position had changed. We did the same experiment with humans but they noticed nothing new, until it was pointed out (Kiley-Worthington unpublished experiment 2009 [95]). 

Voluntary or instrumental learning is when a stimulus becomes attached to a particular response as a result of a reinforcement when the response is voluntary. For example, teaching a child or a cow to lift her left leg with the command “lift your left leg” requires the marrying of various cues with the correct responses step by step, but once the stimulus (word) is recognized, the child or animal has a choice: “to do it or not to do it” and may get it right or wrong. Therefore, performing a voluntary act *requires decisions and choices*: “to do or not to do”. This decision or choice, requires *simple reasoning*: “if I do this I will avoid that or obtain this”, thus, animals that learn voluntarily must take *rational decisions,* at least in the short term. But, voluntary instrumental learning (and probably also Pavlovian conditioning see Thorpe [71]) also involve *anticipating or predicting,* that is “the result of this decision will be this or that”, attached are beliefs: “I believe that if I do this, I will avoid or achieve that”. Then ideas concerning what will or will not happen, and how to accept or avoid this or that are acquired. This is generally called thinking. Something like: “if I do this, I will avoid that and consequently this will happen, but if I do not do this, that will happen, or will it”? Even uncertainty may be involved. The subjects are not thinking in human language but nevertheless, they must have *mental events taking place which denote the conceived situation and it’s possible outcomes* and they make mistakes which confirms that it involves some mental work. 

To learn voluntarily a memory of what happened the last time is necessary. Something like “last time I had to do this to obtain that”, this memory involves beliefs and therefore it involves predictions of events in the future: “last time in this situation, that happened, so it may happen again” (Udell et al. [96]). Having beliefs or ideas of the past and the future has been called “mental time-travel” (Terrace & Metcalfe 2005 [55]) but voluntary learning, because memory is involved, has to involve some ideas about that situation in the past and predictions in the future. This first step in mental time travel has been largely ignored by the researchers today who argue that “the ability to travel mentally in time constitutes a discontinuity between humans and other animals” (Suddendorf & Cornwallis [97], Tulving 2005 [98], but what length of time is to be regarded as “time travel”? Predicting or remembering in years may not be what non human mammals do, but, perhaps they could learn. For example, adult mammals who are familiar with seasonal changes, know both physiologically and behaviourally when winter is coming, and some even plan and store food for this (e.g., squirrels), or migrate to areas where there is food.

The easiest way in which incidences may be remembered is by remembering particular episodes: episodic memory: “I remember/visualize what happened last time I was in exactly this situation”. Episodic memory is defined as the memory of autobiographical events, the collection of past personal experiences that occurred at a particular time and place (Table 1). It is believed by many cognitive scientists, that episodic memory has to be proved to exist (Tulving 2005 [98], Suddendorf 1994 [99]. If non-humans do not have episodic memory then they do not have past personal experiences. Surely, if the individual has a memory (which, to learn to do voluntary actions he has to have), he will remember the event and the situation surrounding it: that is the previous episode? He will not react with a reflex (Table 1) when decisions have to be made, (unless such behaviour has been previously learnt and become a habit: see below). Why would non-humans/non-primate mammals not be able to remember and recognize that particular situation and episode? If they cannot, why do they behave as they do when placed in the same situation? For example, a horse who had a frightening experience when entering a trailer refuses to enter the same trailer later, but entered a different one in a different place. Even if this is described as “episodic-like memory” (Clayton 1998) [100], all who learn voluntary actions must have it (de Waal [32], for further discussion and primate examples), surely, this is a simpler explanation? 

A more important question than “proving” that mammals have episodic memory, is to ask to what degree and in what way, does the situation have to be similar before the same response is obtained? This may depend more on the individual’s past experience than a simple rule of thumb. For example, when a horse has been frightened by going through a particular gate, when does he recognize the same width, size, footing etc. of another gate as similar and therefore dangerous, and when is the gate sufficiently different for him not to worry about passing through? Such questions bring us nearer understanding his “concept” of gate, or trailer, (e.g., Table 1), and the types of judgements he makes concerning whether he can pass through or not, both interesting questions that need answers.

The establishment of habits is one of the reasons why there is confusion concerning the necessary mental attributes associated with voluntary learning. Habits may be established after a behaviour has been learnt consciously and voluntarily when it is frequently repeated. When it becomes a “habit”, then it can be performed with no conscious decision making (e.g., Killcross & Corteau [101], Neal et al. [102]). This has obvious advantages; it liberates the attention and consciousness to be directed elsewhere while the habit is being performed. But, to become a habit, the behaviour has first to be learnt voluntarily with full attention. Habits can be brought back to conscious awareness and changed when different events occur and different decisions are required, although this may require much “motivation” (Table 1: Wanting).

Memories are not indelibly inscribed in the brain since they are changeable (Table 1). In different individuals or at different times when the individual plays the memory back, it may change, and become imaginary; thus, the mind changes memory which then becomes imagination (Table 1). Because of this changeability, if one exists, the other does too. One form of imagination is dreaming which is identified by rapid eye movement sleep. Human and non-human mammals often move in their sleep, or make non-vocal respiratory noises, such as sighing, coughing, or groaning which may be “reflexes” performed without mental event. But, when vocalisations are made during sleep, there must be a mental event, “a dream” because vocalisations are only made to convey a message voluntarily to another. Many mammals vocalize when asleep (e.g., dogs growling, barking, whining, horses, nickering, squealing and neighing: (personal observations 1974–2017). Such emotional memory has been found to be enhanced across sleep intervals with REM sleep (Wagner & Born. [103]), more questions for research. 

### 3.4. Ecological Knowledge

All mammals have to acquire ecological knowledge to survive: what to eat, where to find it, how to catch it. If herbivores, they must become good “*natural botanists*”. If predators, “*natural zoologist*” to know who, when, where and how to hunt. All must become “*natural ethologists*” to identify other species behaviour, know their prey or predator and what he may do next. They must learn when, how, where, if, who to avoid, where to hide, when to run by reading the predator’s feelings/intentions from his behavior. For example many antelope will graze very close to lions when the lions are fully fed and sleepy, but when they are awake, hungry and hunting, the gazelles make themselves scarce (Thurfjell et al. [79] and Kiley-Worthington [85]). 

All mammals must learn to be “*natural geographers*” to know their way around their home areas, find shelter, water, food, comfort. They must become “*natural meteorologists*” to know what the weather might do to avoid sever winds, avalanches, shelter from storms or sun; “*natural geologist*” to know where and when to dig, climb, walk (Kiley-Worthington 2000 [85] further discussion). They must become “*elementary physicists*”, for example understand something about gravity: stones fall down hill, how to lift, move, break objects or avoid them, to move at speed, catch balls or prey, how and when to leap from one branch to another if arboreal. They must learn how to balance on their legs when they move, practice the different gaits and how to twist and turn when chased. All this is learnt, although some precocial species may learn it fast very soon after birth. For example precocial foals learn to stand and walk usually within 3 h of birth, but to perform some of their gaits, 24 h may be needed ([8]). What is learnt depends on the environment in which they live (review: Avital & Jablonka [82]). But, once it is learnt, it can become a “habit” which does not require attention in its doing (Yin et al. [8,104], see below). 

The amount and variation of the ecological knowledge that every mammal has to acquire, if he is to survive and reproduce, has rarely been itemized for one species to date (although see de Waal [32] for primates & Kiley-Worthington [8,85] for equines and elephants). How this knowledge differs will dependent on: (i) the species; (ii) his lifestyle and (iii) his habitat. It must also be born in mind that different species who live in the same habitat such as humans, dogs and horses living in cities, may have more ecological (and social knowledge) in common than they have with other members of their own species who live in very different environments such as wild or remote areas. But, it is always possible for any mammalian individual to acquire new knowledge, although some individuals or species may find this easier than others, and established habits are hard to unlearn (e.g., Neal et al. [102]). 

### 3.5. Social Life and the Social Contract

From their first moments out of the womb, mammals also start to learn social behaviour and it’s rules. They must learn to be “*natural sociologists/psychologists*” (Thornton etc. [69] and Jolly [105]). First: who their mother is and when, where and how to suckle, who their siblings are and how to behave towards them. This is done by reading others’ feelings to know what they might do from observing their different and sometimes erratic behaviours. This cannot be “genetically programmed”. Young mammals, as well as adults, make mistakes in their readings of intentions as do children.

As they grow up all mammals learn their “*social contract*”, that is the rules of that society (Rousseau [13]). For example, they learn to treat young differently from adults, females from males. They must recognize who is a member of their group and which individual to approach, who to follow, who to avoid. They also acquire knowledge about other species they encounter and can form social bonds with other species which may have different social contracts, which they may learn (e.g., dogs, cats, wolves, foxes, horses, elephants, rhino with humans, or between each other, for example buffalo with zebra in the wild (e.g., Prins [77]), and these may differ (e.g., the different relationships cats and dogs have when living in different households (Johnson [42], Cooper Ashton [51], Kiley-Worthington & Rendle [70] and Horowitz [106]).

The social homologies that all species of mammal must have to breed and successfully raise their young are: (1) to learn to recognize their mother and when to suckle (2) recognize other members of their own species; (3) To discriminate males, females, and young from adults; and (4) to recognize individuals with whom they associate; (5) To recognize different species and learn something about their behaviours. Mammals raise their own young, so even solitary ones, have a level of social life and must read *intentions* and mood or feelings of others: is he going to attack or avoid me, be nice or nasty, playful or cross. To do this, each must *recognize that others are beings*, *with bodies and minds: feelings and intentions.*

### 3.6. Awareness of Others Having Minds

Consequently, all mammals have to have a theory of mind, (Table 1), that is recognize that others have beliefs, desires, intentions. This awareness has to be a *baseline mental aptitude* for all live normal mammals. Some species or individuals, may predict what others will do better than others (e.g., some horses predict humans behaviour better than some humans of horses: Kiley-Worthington 2011 [8]) but *all mammals must have a theory of mind because they are living sentient communicating body/mind social beings who recognize others and know something about the emotions/feelings/intents of the other.*

If mammalian society has no social rules (a social contract) then how can it have an organization, where each knows who to approach and when, who to avoid, and how to behave to remain in the group? The study of animal “social networks” (Krause & Croft 2015 [107]) takes this for granted, yet no one to my knowledge, has mentioned that social networking inevitably requires a theory of mind [15]. The particular social contract is superimposed on awareness of intentions of others, and varies as a result of the instinctive tendencies of the species, the environment and life time experiences. This results in different *cultures and traditions* (e.g., Avital & Jablonka [82] and Bonner [83]). 

The current belief among many cognitive ethologists, taking the reductionist approach, is that non human non-primates do not have a theory of mind until it is experimentally proven (e.g., Terrace & Metcalf 2005 [10,55], Tomesello 2009 [108] and Penn & Povinelli [109]). Many have shown that the species they work with has social rules, thus a social contract, but they deny them a “theory of mind”, that is that they must be aware of others and their intentions. This pre-supposes that unless proven otherwise, non-human mammals are all suffering from something like autism (a mental condition characterized by great difficulty in communicating and forming relationships with others). There may, indeed, be some autistic individuals who are unaware of others’ intentions, but normal mammals do *not* ignore others’ intentions, feelings or the social norms of that society, demonstrated by the fact they all have some social organization. 

Although it may be interesting to test whether other species of mammal shows compassion or empathy, (Premack and Woodruff [15] and many since) such demonstrations are not necessary for having a theory of mind. Just an awareness of another’s intentions and feeling is enough (Kiley-Worthington 2005 [9], Midgley [18,75,110], de Waal [32]). It can be the case that one is aware what another mammal is feeling, but this does not mean that one has to be sympathetic with that feeling or act compassionately; having a theory of mind is not dependent on acting sympathetically or compassionately at least until it is redefined. 

One of the major evolutionary questions is why individuals are social. The reasons usually given are: (a) protection from predators; (b) finding mates. But probably the most important reason for social living among mammals is *to pass on important ecological information by social learning*. Instead of every individual having to acquire all the information about the environment that he needs to survive by trial and error; in a sense “inventing the wheel” he can acquire information about the environment and social norms by observational and social learning from others’ mistakes, as well as correct choices. Many of these learnt behaviours may become habits but initially, particularly the “whats” and “hows” were learnt voluntarily when choices and decisions had to be made and others’ intentions recognized, because each is aware that the other is a “being”. 

Social facilitation or social synchronization (e.g., Rook & Penning, [111], Conradt & Roper [112]) occur frequently in mammals and become habits. What has become known as genuine “imitation” (Table 1, Yin and Call [104,113] pp. 322–324) has been less often reported. Imitation has been shown to be an important tool in learning and directing attention in a range of species including human infants, primates (de Waal 2016 [32]), ungulates and elephants [8], canids: (Bekoff [114]) and between species (e.g., horses, cattle, dogs, elephants & guanacos who learn to imitate a human teacher (Kiley-Worthington & Randle 1998 [43]). Dorey ([115]) maintains elephants were not able to imitate humans doing novel actions (Video to the contrary is available [70]). Imitation inter- and intra-species may be much more common than believed. 

### 3.7. Self-Awareness

Awareness of self has been the subject of discussion and experimentation since (Gallop 1982 & [116]) used a mirror to test whether chimps were self-aware, but there are some inappropriate experimental procedures and conclusions concerning this issue (see page 6). Why every species should know their own reflection so well that any change to it will cause them to touch the changed area, or why it is assumed that all mammals will be either familiar or interested in what their face looks like and why this should be a test of their “self-awareness” remains a mystery. When adult tribal Africans who had not seen photographs before were given pictures of themselves, they were initially unable to recognize themselves; does this mean that they were not self aware? (personal observation 1950 with the tribal Maasi Morans in the Serengeti, Tanzania). More recent tests have been invented for self awareness (see Call [10,113] for review), but even these, do not always take into account that mammals must be aware of their own bodies, because they feel them. They feel pain and know where the pain is: “my foot”, “my back. They cannot feel unless they are aware that it is them that is feeling. They can also see at least parts of their bodies, so they are aware that they have a body and that their being (body and mind) behaves, has body functions like urinating and defaecating or can pick up a thorn, itch, be warm or cold. They also have desires and intentions (eating, drinking approaching, avoiding) and make choices (what to eat, where to find it, etc.). This is all part of “my being”, “my deciding”. An awareness of their own body/mind being, is therefore a baseline mammalian characteristic, because otherwise they could not feel. 

Whether they ponder or reflect on their feelings and themselves and decisions they have made, is not the same question as whether they are aware that they exist and that this foot/hoof/tongue is mine because I can feel it hurting (Stamp-Dawkins [117]). Questions such as whether that species shows compassion and empathy are interesting, but if measured by action, they reflect more the ability of that species to manipulate things rather than what they may be feeling. Primates and elephants are manipulators, but this does not tell us whether they are more or less empathetic than a cow or a horse who cannot lift a youngster out of the mud. If “awareness of others” is restricted to demonstrations of physically manipulate and helping others, this tells us little about self-awareness.

### 3.8. Moral Agency

Recognizing moral agency (Table 1) in non-humans is not new. Mammals for centuries were dismissed from being moral agents, but, in 17th century Catholic France) pigs and rats were held responsible for their actions in courts of law (Evans [118]). Mammals have a social contract with social rules, such as, “do not kill and eat infants of your own species” or, “do not kill another who is submissive and not threatening you”, or “learn your own and others’ roles” in the society (e.g., Ricci-Bonot & Kiley-Worthington 2017 [119]). That is, they learn the rights and wrongs of living in their social group. Debates concerning what appertains to moral agency have continued since Aristotle, and probably before. Recent philosophers have reviewed whether non humans are moral agents using moral theory (Rowlands [120,121], Shapiro [122]), but since all mammals have a social contract, and know the rights and wrongs of their society, it would seem that there is little doubt using this definition. 

Many pets and working animals learn the rights and wrongs of what they are to do when living with humans. They exercise choice: to do the right or the wrong thing. Hence they are moral agents who can be held responsible for their actions like children, not just beings of moral consideration or moral standing.

It is clear that many of us treat our dogs as moral agents: the individual is responsible for his actions and his desires are recognized and may be considered by others. They also respond as moral agents as children do. They learn the rights and wrongs of their behavior and act accordingly, and may treat us as moral agents (Kiley-Worthington 2005 [9] and Horowitz 2010 [106]). Feeling shame or guilt does not define moral agency although whether non-humans feel it is an interesting question. Then again, criminals may not feel either shame or guilt, but they are considered moral agents. 

To summarize, to be a moral agent, *the individual knows that s/he has or has not obeyed the rules of the society that he has learnt*, often because of reward, negative reinforcement, or punishment. As a result, mammals make decisions on how to act based on the social rules that they have learnt; therefore are moral agents. They do not debate this, as far as we know, but there are many humans, all of whom are considered moral agents, who do not either. In human society, moral agency may become more complex, but fundamentally it is the result of being social and having a social contract.

### 3.9. Aesthetic Sense

Whether animals have an aesthetic sense has only recently re-emerged since (Darwin [26], Kiley-Worthington [8,85], Watanabe [123] and Welsch [124]). A sense of aesthetics at its simplest is that certain sensory stimulations, whether visual, auditory, olfactory, taste or tactile, are liked (by the subject) and others are not (Cooper [6]). Aesthetics is based on feelings. Species and individuals show likes and dislikes of many things. For example, both humans and horses generally like views and will often go to areas where they can have them in preference even to eating. This is an “instinctive tendency” that has evolved to help survival (they are both species that need to spot predators). Of course such an aesthetic sense has an evolutionary background, but *in the proximal world it is still a choice*; and they choose to look at the view or not, depending on their own experiences. As a result, there are individual variations in likes and dislike which are the result of the individuals innate tendencies moulded by his lifetime experiences. One individual may learn to like something that others in their group also like or not. 

Non-humans do not, as far as we know, discuss aesthetic appreciation and beauty, but they do often follow aesthetic fashions, for example where to sit or stand and stare, a nice place to sleep, a nice view, a nice taste or feeling, or a nice smell, or noise (dairy cows have been shown to let more milk down when familiar music that they apparently like is played, and stop milk let down when threatening loud noises are made: Kiley-Worthington unpublished experiments 1978). Interesting philosophical, ethological or evolutionary questions can now be asked on what aesthetic sense does that species (or individual) have particularly well developed. It is possible that if humans appreciate in more depth different aesthetic values, they could enrich their own.

Proving any of the above mental attributes in any non-human mammal does not further our understanding of that species cognition because they define what a mammal is. Logic dictates that they all must have them as part of the mammalian package, although some species may or may not have specialized in one or the other.

A recognition of these basic mental homologies in mammals is the starting point for a serious appraisal of general mammalian epistemology (Table 1) Thereafter studies of the particular being of that species, his/her body, and the way it works, habitat, lifestyle, consequent instinctive tendencies, knowledge and experiences, all critically assessed, will point to that species general epistemology. Let us hope that this is assembled before more species become extinct, (e.g., rhinos). 

Some have attempted to outline the epistemology of some species (some primates de Waal [32] and others), some cetaceans (Whitehead & Rendell [125] and others), elephants (Kiley-Worthington [85], Moss [126], Shand 1991 [127]) dogs (Horowitz [106] & others), horses (Kiley-Worthington [85], Rees [128] & others), but the approaches are different, and often the mammalian mental homologies are not recognized. Putting together all that is known and critically assessing it from any discipline, results in nine mental homologies that define a mammal (Table 2). It is possible that more will be discovered in the next years, provided the research goes forward. This is the first step. None of this is new. What is new is acknowledging these mental homologies and the other mental aptitudes related to them and leads to more interesting research questions (suggestions are given in Table 2). 

All these mental skills are developed in different ways and to different levels as a result of the lifestyle and being of the different species, they are part of being a mammal. Once recognized, other dependent mental attributes are evident. 

The recognition of the mental aptitudes that define a mammal, is the first step to understanding an individual’s subjectivity/epistemology (knowledge and view on the world & personhood). The secondly step is a critical assessment of how these are specialized or modified in a particular species because of his body & mind. The third step is the assessment of how that individual of that species, develops and modifies his mammalian homologies as a result of his individual genetic constitution and lifetime experiences. This three tier approach (Table 3) is called Conditional Anthropomorphism (see Fisher [49] and Burghardt [8,50]).

There are both pure and applied scientific and philosophical reasons why such a study is important:

(1) By recognizing mammalian mental homologies, we can escape from asking irrelevant questions and begin to unravel non-human mammals mental similarities and differences with greater speed and rationality.
(2) Further knowledge has implications for the family, species and individuals’ welfare and needs. Only in this way can we begin to provide *a life of quality* for species or individuals whether in nature reserves, any form of captivity, domestic, on farms, pets, used for recreation, human therapy or working.
(3) Recognising non human mental homologies leads to recognizing that all mammals have other mental dependent attributes for example: reasoning, anticipating and predicting, beliefs, mental time travel, episodic memory, making judgments, having concepts and imagination and acquiring a vast amount of ecological and social information.
(4) It raises many questions concerning different worlds, free will and species and individual epistemology.
(5) An understanding of another species or individual’s epistemology makes us think in new and different ways and appreciate different things. It can mould our world view and enrich our lives. We might learn something about how to live or die better, and could solve some of the worlds threatening problems created by the present homocentric world view.

We no longer need to throw up our hands and assume that the mental world of another species is impossible to comprehend or even imagine, (Nagel 1974 [129]). We need now to recognize our mammalian commonalities unclouded by historical beliefs as well as species differences. 

## 4. Conclusions

The study of animal minds and cognition has been greatly fettered by historical and cultural beliefs and the rise of humanism (Gray [130]). But, this has not always been the case. When human populations were in contact with non-human mammals daily, they mutually acquired knowledge about each others’ mental attributes based on common mental abilities/homologies. Today, this folk or common sense knowledge is ignored by scientists and some philosophers, or denigrated to the status of “not science” and therefore “not knowledge”. If we are to advance in our understanding of different species and individuals’ cognition and epistemology, all relevant information must be accumulated from folk knowledge, the behavioural sciences and philosophy of mind. All must be critically assessed so that dogmas and pre-conceptions can be unraveled. The first step is to recognize the mental homologies that define mammals. 

We are in the curious position today, that species we have relatively little information about such as cetaceans, primates and elephants (which have only been studied for some decades and no one lives with), are believed to be more mentally able than any animal on our farm or by our fireside, although things may be changing as dog cognition (mans’ best friend) is now receiving attention (e.g., Udell [96], Horowitz 2010 [106], Gacsi et al. [131], Ákos et al. [132]). But, there are also other beliefs which colour current cognitive science arising from a profound belief in experimental proof. Conclusions concerning the mental inabilities of non-human mammals are often based on assumptions, negative experimental results, inappropriate tests and a belief in humans mental superior in almost all respects. 

In the light of the driving force of emotions, simplicity, logic, folk knowledge from centuries of contact, detailed observational studies, inter-species teaching and emotional contact with other species, the stage is now set for study of meta-cognition of many mammalian species. The first step here, is to identify the mental homologies that define mammals using simple logical arguments and recent relevant research from any discipline, rather than relying on opinions or only experiments. 

Once these mental homologies are recognized, a recognition that all mammals must also have some other mental attributes follows. These include their abilities to make choices and decisions, to rationally reason and have beliefs. Voluntary learning shows that they have to anticipate and predict, make judgements and developing concepts, remember and imagine, have episodic memory and learn to imitate each other. All of this means that they have ideas, thoughts, and dreams and can “travel in time” that is have some idea of both the past and the future, and to know what is remembered (Hampton 2001 [133]).

Because they are sentient: feel, they are all conscious of being awake and in the world, and self conscious of their own body/mind beings. To survive and breed, they learn about their world and therefore have experiences and acquire knowledge about the environment and the social norms and have traditions and cultures. They have a social contract and therefore are moral agents, and they like and dislike things, therefore, have a simple aesthetic sense. All of this indicates that they have a subjective point of view: an epistemology and a personhood. The driving force behind these mental homologies appears to be sentience/feelings/emotions. Of course, different species develop one or other of these mental attributes differently, but all mammals have them. As a result of recognizing these mental homologies in mammals it is not clear why, or if, humans mental attributes and trivial interests, should always trump those of non-humans. 

I hope this paper will stimulate further discussion about the beliefs that colour the opinions and science of cognitive and animal welfare scientists, philosophers and animal welfare activitists debates. To ignore what defines a mammal’s whole being, makes little sense and throws out the baby with the bathwater if we want to find out more about their epistemology. 

We should give the other mammal the benefit of any doubt that we may have, as we have no time left. We are loosing mammals fast. It is predicted that by 2050 there will be a 68% extinction of animal species from 1970 (Tilman et al. [134]). They are vanishing before we even recognize their beings, and with them goes their epistemological uniqueness which could enrich our own homocentric lives. Suggestions for future relevant research in this regard have been made, and the future is wide open for observational, laboratory work and critically assessed experiences in order to discover more about mammalian meta-cognition. 

The way forward is to develop a multi-disciplinary approach to understanding another’s epistemology, we have suggested Conditional Anthropomorphism. This paper addresses the first stage: what common mental attributes do all mammals have.

## Figures and Tables

**Table 1:** The definitions of the terms used in this article.

A Being	A Living Individual Consisting of a Combined Body and Mind.
**Aesthetic Sense**	This is used in the Kantian sense of being effected entirely by the subjectivity of the subject, thus, if an individual likes/appreciates some things and dislikes others he has some aesthetic sense. This may relate to any of the senses Cooper 1992 [6].
**Choice**	A voluntarily choosing between two or more alternatives of action requires mental action.
**Belief**	An attitude involving the recognition or acceptance of something as real.
**Cladistics**	Study of members of a group share a common evolutionary history, and are “closely related. Recognized by sharing unique features which were not present in distant ancestors. These shared derived characteristics are called synapomorphies (Guralmick 2014 [7]).
**Concept**	Identification of a class of objects.
**Conditional Anthropomorphism**	A recognition of mammals mental homologies by critically assessed some mental characteristics of human mammals (anthropomorphism). The different species mind/body differences are supermimosed (conditional). The individual’s differences as a result of his genetic make up and past experiences then considered. An approach towards an individual’s or species epistemology: knowledge of the world. (Kiley-Worthington 2011 [8,9]).
**Conscious**	Awake, feeling and consequently aware of being in the world.
**Decisions**	Making a voluntary choice to do one thing or another which involves mental events.
**Dualism**	A belief that the mind is separated from the body.
**Episodic Memory**	Remembering a particular event, what, where and when it happened Wynne & Udell 2013 [10].
**Epistemology**	The study of knowledge, used to denote the world view gathered from the knowledge & experiences that that species or individual has.
**Folk Belief**	A general belief about another species/or thing that is the result of preconceptions that is beliefs which have not been critically assessed.
**Folk Knowledge**	Critically assessed knowledge acquired by ordinary people, non specialists in cognition.
**homeothermic**	Warm blooded animals.
**homology**	A similarity in structure, origin and development indicative of a common ancestry.
**Idea**	Some immediate object of the mind which it perceives and has before it (Locke 1689 [11]).
**Imagination**	A mental faculty forming images of external objects not present to the senses.
**Imitation**	The imitator performs a novel act as a result of seeing it performed (Wynne & Udell 2013) [10].
**Linnean Systematics**	Linneaus’s classification of species. He sorted plants and animals into their different phyla, genuses and species on the basis of the degree of their structural similarities Grove and Newell 1942 [2].
**Memory**	Recalling in the mind, recollect, not forgetting.
**Mental Time Travel**	Remembering things in the passed and predicting things in the future Wynne & Udell 2013 [10].
**Mind**	The organized totality of mental structures and processes, illustrated by making choices and decisions and having feelings that are felt.
**Monism**	A belief that the mind and the body are one, united to make the individual being.
**Motivation**	Term employed generally for the phenomena involved in the operation of incentives or drives. In common English “wanting to”: a feeling.
**Moral Agency**	A moral agent is an individual who knows the social contract of his society, therefore knows right from wrong in that society. S/he is a subject, and has a theory of mind (knows that others have minds with desires and needs).
**Objectivity**	The belief that there are certain truths that will always remain true, what ever the thoughts or desires of others.
**Personhood**	Having an individuality of combined body-mind being.
**Reflex**	An involuntary mechanical or autonomic response performed without mental events.
**Self-Awareness**	As a base level: an awareness of one’s own body, feelings and existence.
**Sentient**	A being that has feelings and emotions and therefore can suffer.
**Sense of Justice**	Is when the individual recognises that he or another has or has not obeyed the rules of the society: the social contract, and recognizes the cause of the reward or punishment that follows.
**Social Contract**	An agreement between individuals in which some personal liberties are freely surrendered for the advantages of having a well organized society (Plato: the Republic) [12]. Having and knowing the social rules of the society Rousseau 1762 [13].
**Subconscious or Unconscious**	mental processes occurring outside the personal awareness of the individual subjectivity. Being a subject in the world with an individual point of view, the result in part of the individuals passed experience (learning/culture).
**Synapomorphy**	A derived character of a clade that has been inherited from a common ancestor and distinguishes the clade from other potentially related organisms is called a “synapomorphy” a shared character that sets the clade apart. (Barton et al., 2007 [14]).
**Theory of Mind**	Recognizing that another has a mind, feelings, desires and intentions. (Premack and Woodruff [15]).
**Thinking**	A course or train of ideas.

**Table 2:** Mammalian Mental Homologies. The baseline mental skills that define mammals.

Mammals have “innate/instinctive tendencies” to do particular things which are not inflexible. Each species has certain different “*instinctive tendencies*” *but these are moulded by their lifetime experiences.* Future research should look at *how, when where and why they are moulded.*
Mammals are sentient, that is they feel or experience emotions and have a mind to do this with. Because mammals feel; they are conscious of that feeling & are therefore *aware of being in in the world.* They have feelings in common which are communicated both within and between species. Further research on outlining *how to measure and assess* emotions is urgent for improving welfare, and the different emotions different species experience.
They learn. They *show flexibility of behaviour which moulds their instinctive tendencies.* They learn by Pavlovian and Instrumental Conditioning, Associative and Silent/cognitive learning and thus *acquire knowledge.* Future learning research should concentrate on the knowledge that a species generally has as well as find out what else he can learn.
To survive, they must acquire information. They acquire *ecological knowledge:* how, where, what, when to lie down, to stand/walk/suckle/eat/drink/shelter/find their way around, etc. This information may be different in different environments, which leads to *different cultures* with different ecological knowledge. *What* this is and how their cultures differ is a research question.
They have social tendencies and must become *good natural sociologists*. They have to learn the social rules of that group: the *social contract*. More detailed knowledge of different social networks in different species is needed.
They have to be aware that another has feelings, intentions, knowledge, makes choices and decisions, that is they are aware that others have body/mind “beings”, called *having a* “*theory of mind*”. This i*s a necessity mental ability of any normal mammal.* How good different species or individuals are at this is a research question.
Mammals are self aware of their own body/mind being. *They know their own body is theirs*, whether it is a leg, arm, stomach etc. By imitating others particular movements, they demonstrate this. They also *have their own feelings*, *memories and experiences*; *to this degree they are self aware.* Further research on interspecies imitation and whether non humans can be reflective consciousness are research questions.
Mammals are, simple moral agents since they have a social contract (rules of their society) and *know the rights and wrongs of what they must do to stay in that society*. Whether they feel shame or guilt is a future research question.
Mammals have a simple aesthetic sense, since they are sentient and *they like and choose some things and dislike and avoid others*. What different species or individuals particularly like and dislike and why, are research questions.

**Table 3:** The Conditional Anthropomorphic assessment of another mammal’s epistemology.

Anthropomorphic. Body/Mind homologies. A recognition of homologies in body *and mind* which define mammals. Since we are human mammals, we should initially, assess others from our own standpoint with homologous body parts and mental abilities.
This is Conditional on: (2a) The species peculiarities superimposed on these homologies of body and mind. A study of each species differences in body and mind (e.g., size, speed of movement, morphology, lifestyle & sensory abilities and their resulting “instinctive tendencies” which define the species. But, the species behaviour can be moulded by the environment; (2b) The Individual’s epistemology which is the result of his individual innate genetic tendencies consisting of his mammalian homologies and his species peculiarities *moulded by his lifetime experiences in body and mind.* His individual experiences will differ from other members of his own species. His lifetime experienced should be studied closely to have at an idea of that individual’s personhood.
